# Anti-*Helicobacter pylori* Activity of *Artemisia ludoviciana* subsp. *mexicana* and Two of Its Bioactive Components, Estafiatin and Eupatilin

**DOI:** 10.3390/molecules26123654

**Published:** 2021-06-15

**Authors:** Juan Francisco Palacios-Espinosa, Pablo Noé Núñez-Aragón, Erika Gomez-Chang, Edelmira Linares, Robert Bye, Irma Romero

**Affiliations:** 1 Departamento de Sistemas Biológicos, División de Ciencias Biológicas y de la Salud, Universidad Autónoma Metropolitana-Xochimilco (UAM-X), Ciudad de México 04960, Mexico; jpalacios@correo.xoc.uam.mx; 2 Departamento de Bioquímica, Facultad de Medicina, Universidad Nacional Autónoma de México (UNAM), Ciudad Universitaria, Ciudad de México 04510, Mexico; pablo.nunez@uaem.mx (P.N.N.-A.); ergoc@bq.unam.mx (E.G.-C.); 3 Jardín Botánico, Instituto de Biología, Universidad Nacional Autónoma de México (UNAM), Ciudad Universitaria, Ciudad de México 04510, Mexico; mazari@ib.unam.mx (E.L.); bye.robert@gmail.com (R.B.)

**Keywords:** *Artemisia ludoviciana*, estafiatin, eupatilin, *Helicobacter pylori*, gastroprotective, anti-inflammatory

## Abstract

*Artemisia ludoviciana* subsp. *mexicana* has been traditionally used for the treatment of digestive ailments such as gastritis, whose main etiological agent is *Helicobacter pylori*. In a previous screening study, the aqueous extract exhibited a good in vitro anti-*H. pylori* activity. With the aim of determining the efficacy of this species as a treatment for *H. pylori* related diseases and finding bioactive compounds, its aqueous extract was subjected to solvent partitioning and the fractions obtained were tested for their in vitro anti-*H. pylori* effect, as well as for their in vivo gastroprotective and anti-inflammatory activities. The aqueous extract showed a MIC = 250 µg/mL. No acute toxicity was induced in mice. A gastroprotection of 69.8 ± 3.8%, as well as anti-inflammatory effects of 47.6 ± 12.4% and 38.8 ± 10.2% (by oral and topical administration, respectively), were attained. Estafiatin and eupatilin were isolated and exhibited anti-*H. pylori* activity with MBCs of 15.6 and 31.2 µg/mL, respectively. The finding that *A. ludoviciana* aqueous extract has significant anti-*H. pylori*, gastroprotective and anti-inflammatory activities is a relevant contribution to the ethnopharmacological knowledge of this species. This work is the first report about the in vivo gastroprotective activity of *A. ludoviciana* and the anti-*H. pylori* activity of eupatilin and estafiatin.

## 1. Introduction

*Helicobacter pylori* is a Gram-negative bacterium that infects more than half of the world’s population. A recent systematic review and meta-analysis on its global prevalence demonstrated that *H. pylori* infection has an overall prevalence of 44.3% and, in some countries, the estimated rate is as high as 89.7%. Several factors like hygienic conditions, urbanization, and socioeconomic status seem to dictate the prevalence of infection between countries [1]. *H. pylori* is an important etiological agent of severe gastroduodenal pathologies, including gastric and duodenal ulcers, adenocarcinoma and gastric lymphoma [2]. In addition, *H. pylori* infection has also been associated with several extragastric complications such as hematologic, cardiovascular, and neurodegenerative diseases [3]. Although the incidence of *H. pylori* infection is declining in many countries, the incidence of gastric cancer persists as a major public health problem. In 2018, it ranked second in the total number of cancer-related deaths in the world (782,685), and 1,033,701 new cases were estimated [4].

*H. pylori* possesses several well-developed mechanisms such as an acid acclimation system, adhesins, chemosensory-directed motility, the multifunctional toxin VacA, and the cag pathogenicity island, which make the bacterium able to survive and proliferate in the harsh environment of the human stomach [5]. It has been demonstrated that, in addition to environmental factors, the bacterium and host polymorphic genetic factors play an important role in the final outcome of the infection [6,7]. Conventional eradication therapy combines two antibiotics (amoxicillin and clarithromycin or metronidazole) and a proton pump inhibitor; however, the success of the therapy is limited due to increasing antibiotic resistance rates, high cost, significant side effects and patients’ lack of adherence to current treatments. These issues have given rise to a worldwide demand for new therapeutic agents and the development of novel strategies for the control, prevention, and treatment of *H. pylori* infection [8]. An approach to discover new anti-*H. pylori* agents has been the study of natural products used for the treatment of gastrointestinal illnesses [9,10]. Approximately 80% of people in the world rely on traditional medicine for their health care [11]. In this context, a large number of Mexican medicinal plants have been evaluated for their in vitro anti-*H. pylori* activity and their gastroprotective effect [10,12,13,14]. In particular, *Artemisia ludoviciana* subsp. *mexicana* extracts showed in vitro anti-*H. pylori* activity [12], demonstrating its potential as a source for bioactive compounds.

*A. ludoviciana* subsp. *mexicana* belongs to an infraspecific taxon of the species *Artemisia ludoviciana* Nutt. in the genus *Artemisia* (family Asteraceae), which has a further 39 synonyms [15]. This species has been medicinally used in Mexico since pre-Columbian times, and is popularly known by names such as “estafiate”, “istafiate” “ambfe”, “ajenjo” and “artemisia”, among others. *A. ludoviciana* has been traditionally used in Mexico for the treatment of digestive, hepatic and biliary diseases. For instance, an infusion or decoction from the aerial parts of the plant is used for the treatment of gastric discomfort, diarrhea, gastritis, intestinal pain, parasites, vomiting and poor appetite [16]. Alcoholic extracts of aerial parts are applied to the skin as liniments or poultices for pain and inflammation, while plant inhalation is used for respiratory illnesses like colds and bronchitis, as well as for throat and head sores [17,18,19]. Also, antidiabetic activities have recently been reported [20]. Moreover, it has been reported that, in Mexico, this plant is frequently accepted and prescribed for stomach ache, colitis and menstrual colic by primary health care professionals [21]. The aerial parts of *A. ludoviciana* subsp. *mexicana* have been exhaustively investigated phytochemically and pharmacologically. Several studies regarding the chemical constituents of *A. ludoviciana* have led to the identification of more than 70 compounds. The main groups are represented by monoterpenes (e.g., camphor, limonene), sesquiterpene lactones (e.g., estafiatin and ludovicin), and flavonoids (e.g., eupatilin and jaceosidin) [20,22,23,24,25]. With regard to its biological effects, antibacterial [12,25,26,27,28,29] and antiprotozoal activities have been documented [30,31,32]. In addition, other diverse set of pharmacological activities have been described, e.g., antidiarrhoeal [33], and vasorelaxant without spasmolytic effect [34], as well as significant antinociceptive, anti-inflammatory and antihyperalgesic actions [35,36]. Moreover, some studies have revealed the potential of *A. ludoviciana* as a hypoglycemic and antihyperglycemic agent [37].

Referring specifically to *H. pylori* and related diseases, we have previously reported good growth inhibitory activities for *A. ludoviciana* subsp. *mexicana* methanolic and aqueous extracts [12]. Likewise, the above-mentioned anti-inflammatory properties reported for the species could be beneficial in the bacteria-associated diseases that have a strong inflammatory component; however, it is not known whether it may play a gastroprotective role.

Continuing with our investigation to establish the efficacy of *A. ludoviciana* subsp. *mexicana* as a treatment for *H. pylori*-related diseases, in this work, an aqueous extract was subjected to solvent partitioning, and the obtained fractions were tested for their anti-*H. pylori*, gastroprotective and anti-inflammatory activities. Finally, two of the compounds that were isolated were found to be potentially responsible for the antimicrobial activity of the extract.

## 2. Results

### 2.1. Biological Activities of Artemisia ludoviciana Infusion (**ALI**): Anti-H. pylori, Gastroprotective, and Anti-inflammatory Effects

The anti-*H. pylori* activity of *A. ludoviciana* infusion was determined by the broth dilution method. The MIC value obtained was 250 μg/mL.

Since the next step was to determine the gastroprotective and anti-inflammatory activities of **ALI** in vivo, it was necessary to corroborate the safety of its administration in animals. The results obtained by the oral acute toxicity assay in mice using Lorke’s method showed that in both phases of the test, the aqueous extract (**ALI**) provoked neither death nor adverse effects in mice. Furthermore, during the 14-day postobservation period, no behavioral disturbances, signs of toxicity or weight loss were detected in any mice. The estimated LD_50_ was >5000 mg/kg. Therefore, according to Lorke’s criteria, **ALI** has no acute toxic effect in mice.

**Figure 1:**
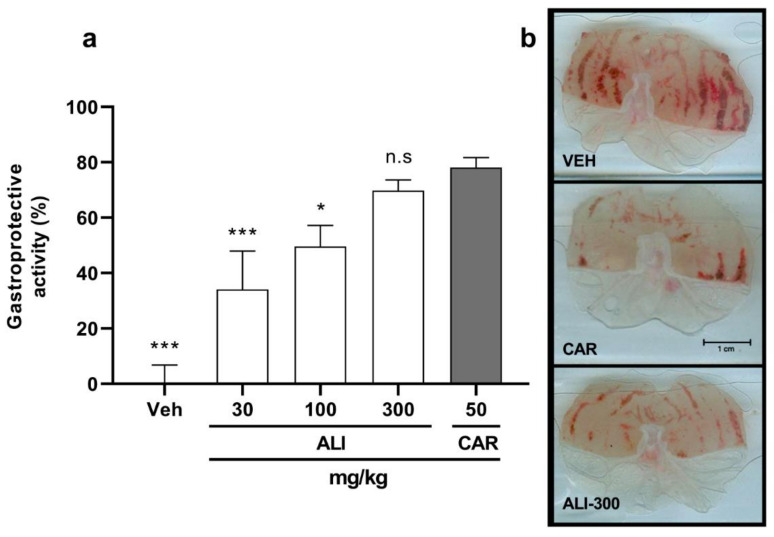
Gastroprotective activity of **ALI** in an ethanol-induced gastric ulcer model in mice. (**a**) Percentage of gastroprotection in the different treatment groups. Each column represents the mean ± SEM (*n* = 7 animals). An ANOVA was performed followed by Dunnet’s test. (**b**) Representative images of mice stomachs from three treatment groups. Macroscopic views of fresh dissected stomachs treated with VEH (upper panel), CAR (central panel) or **ALI**-300 (lower panel). Differences in hemorrhagic streaks can be observed. VEH, isotonic saline solution; CAR, carbenoxolone 50 mg/kg; **ALI**-300, *Artemisia ludoviciana* aqueous extract 300 mg/kg. * *p* < 0.05, *** *p* < 0.001, significant difference vs. CAR. n.s, no significant difference vs. CAR.

Antiulcerogenic activity was evaluated using the acute ethanol-induced gastric ulcer model. Figure 1a shows that the gastroprotective effect of **ALI**, orally administered, was dose dependent and, at the maximal tested dose, i.e., 300 mg/kg, a 69.8 ± 3.8% gastroprotective effect was achieved. This percentage is similar to that obtained with CAR, the reference drug, which was 78.2 ± 3.5% with 50 mg/kg. These estimated ED_50_ for **ALI** was 190.4 mg/kg.

Macroscopic images of representative stomachs are shown in Figure 1b. In the negative control group (VEH), severe damage characterized by the presence of many hemorrhagic streaks, typical of ethanol-induced acute lesions, could be seen. In the case of the positive control stomach treated with CAR (50 mg/kg), a substantially improved appearance of the gastric surface compared to VEH was observed. For instance, the stomach was smoother and only a few scars were visible. Finally, an **ALI** (300 mg/kg) treated animal stomach exhibited few thin damage bands, very similar to those observed in the positive control stomachs. This observation was supported by a statistical analysis, since there were no significant differences between treatments (i.e., CAR and the highest **ALI** dose) in the percentage of gastroprotection (*p* < 0.05).

Additionally, using the xylene-induced ear edema model, a preliminary anti-inflammatory effect of **ALI** was verified by administration via two different routes. Oral (100 mg/kg) and topical (0.5 mg/ear) administration of **ALI** produced similar anti-inflammatory effects (47.6 ± 12.4% and 38.8 ± 10.2, respectively). The anti-inflammatory activities from oral (30 mg/kg) or topical (0.5 mg/ear) indomethacin administration (positive control) were 54.8 ± 4.7% and 61.3 ± 8.1%, respectively.

With the previously obtained results, taking into account that **ALI** has potential gastroprotective and anti-inflammatory effects, we decided to fractionate it in order to simplify the complexity of the extract.

### 2.2. Activity of **ALI** Primary Fractions

**ALI** was subjected to a liquid–liquid extraction process using different solvents, resulting in four primary fractions: dichloromethane (**ALI-1**), ethyl acetate (**ALI-2**), *n*-butanol (**ALI-3**), and a residual extract (**ALI-4**). The yields obtained from each of the fractions are shown in Table 1. Each fraction was screened in vitro for its anti-*H. pylori* activity, and in vivo for its gastroprotective and anti-inflammatory activities. The results are compiled in Table 1, and can also be compared with those of **ALI**.

### 2.3. Eupatilin and Estafiatin as Anti-H. pylori Antibiotics

Taking into account the good anti-*H. pylori* effect of both **ALI-1** and **ALI-2** (Table 1), successive chromatographic separations were carried out in order to find the compounds responsible for the antibacterial activity.

**Figure 2:**
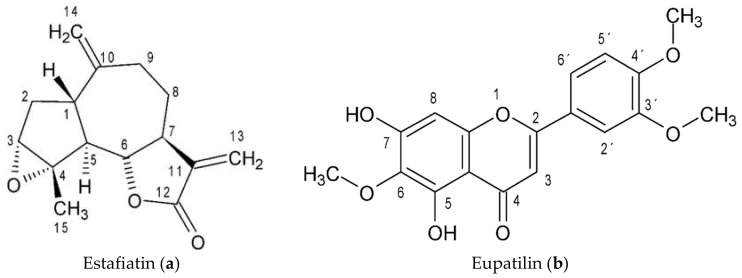
Chemical structures of Estafiatin (**a**) and Eupatilin (**b**).

Through a bio-guided separation of **ALI-1** and **ALI-2**, the compounds estafiatin and eupatilin were obtained (Figure 2), respectively; the identities of the molecules were determined by analyzing their physical and spectroscopic NMR data (Supplementary Materials) and then comparing the data to those reported in previous works. These compounds exhibited a significant inhibitory effect, with MIC values of 15.6 and 31.2 µg/mL, respectively (data not shown). 

**Figure 3:**
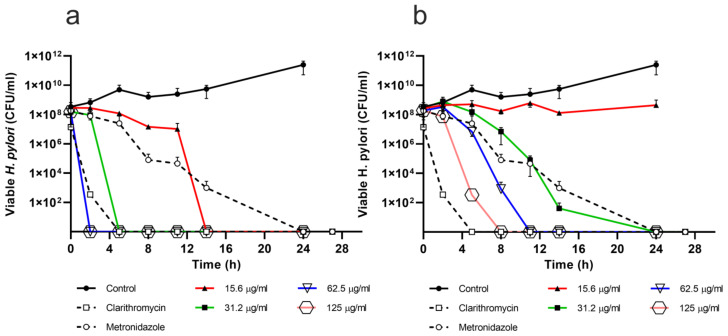
Bactericidal effect of (**a**) estafiatin and (**b**) eupatilin against *Helicobacter pylori*. Bacterial cultures were exposed to different concentrations (15.6 to 125 μg/mL) of (**a**) estafiatin or (**b**) eupatilin. Bacterial viability was assessed by the plate colony count technique at different times of incubation. All the experiments were carried out in triplicate and repeated at least three times. Clarithromycin (0.05 μg/mL) and metronidazole (300 μg/mL) were used as positive controls.

The bactericidal effect of estafiatin and eupatilin is time and dose-dependent (Figure 3). For estafiatin (Figure 3a), the MBC was 15.6 µg/mL, which was attained at 14 h. On the other hand, eupatilin (Figure 3b) showed a MBC of 31.2 µg/mL at 24 h. Metronidazole and clarithromycin, the reference antibiotics, demonstrated bactericidal effects for up to 24 and 2 h with 300 µg/mL and 0.05 µg/mL, respectively.

Urease is one of the most important factors in the pathogenesis mediated by *H. pylori*. This enzyme is involved in the colonization, virulence and survival of the bacteria, and is also a potent immunogen that stimulates a strong immune response. These characteristics make urease a therapeutic target. In order to investigate whether estafiatin and eupatilin could be involved in the activity of this important enzyme, their effect was tested by the colorimetric Berthelot’s method. Eupatilin, at any tested concentration (3.9–125 μg/mL), did not inhibit urease activity compared with the positive control acetohydroxamic acid (IC_50_ = 29.0 μg/mL, and 100% inhibition with 100 μg/mL). In the case of estafiatin at concentrations in the range from 3.9 to 31.2 μg/mL, a slight inhibition was achieved (25–35%); nevertheless, with higher compound concentrations (i.e., 60 and 120 μg/mL), there was no inhibition at all (data not shown).

## 3. Discussion

*Artemisia ludoviciana* subsp. *mexicana* is widely utilized in Mexican traditional medicine, particularly for the treatment of gastrointestinal disorders, as well as other illnesses. With the aim of continuing with the investigations that support the efficacy of Mexican medicinal plants for the treatment against *H. pylori* and its associated diseases, in this work, the anti-*H. pylori*, gastroprotective and anti-inflammatory activities of the aqueous extract and fractions of *Artemisia ludoviciana* subsp. *mexicana* were studied.

In a previous report from our group related to the screening of several plants with anti-*H. pylori* activity, the effect of aqueous and methanol extracts of *A. ludoviciana* aerial parts against *H. pylori* was assessed, and MIC values of 125 μg/mL and 250 μg/mL were reported for the two extracts, respectively [12]. In the present work, the MIC obtained for the aqueous extract (**ALI**) was 250 μg/mL, slightly higher than the previously reported value. The difference may have been due to factors related to the plant origin and/or to the assay used for the MIC determination (agar dilution vs. broth dilution method used here). However, both concentrations can be considered as good to moderately effective [12]. Regarding the MIC values achieved with the reference antibiotics, higher concentrations of **ALI** were required to inhibit the growth of *H. pylori* compared with clarithromycin (0.01 μg/mL) but, with regard to metronidazole (MIC = 300 μg/mL), **ALI** was more potent. At this point, it should be noted that resistance rates to commonly used antibiotics are markedly increasing; hence, the isolation of different and more potent compounds from **ALI** seems to be encouraging. Moreover, it is interesting that **ALI**, at 250 μg/mL, not only had a bacteriostatic effect, but also caused bacterial lysis, therefore exhibiting a bactericidal effect.

In addition to searching for anti-*H pylori* agents, studies conducted on illnesses associated with infections caused by the bacterium are of great relevance. In this sense, the evaluation of **ALI** antiulcer and anti-inflammatory activities is important in order to assess the potential of the extract as an integral therapy for the treatment of *H. pylori*-related diseases. 

With the purpose of ensuring the safety of **ALI** for in vivo experiments, the acute toxicity was evaluated by the accepted Lorke’s model. The test showed that the aqueous extract of the plant was well tolerated and did not provoke any visible toxic effect. Our results are consistent with those already reported by Anaya-Eugenio et al. [37] for an *Artemisia ludoviciana* infusion. Since the infusion of the plant is one of its most popular forms of use in the treatment of gastrointestinal diseases, our results are of great relevance, because they are in accordance with the traditional usage of the plant among the population. Further chronic toxicity studies on the extract will be necessary to rule out any health hazard arising from repeated exposure, and to ensure its safety for future clinical usage. Once a lack of toxicity under acute oral administration had been established, the next step was to evaluate its antiulcer and anti-inflammatory activities. 

Herein, the acute ethanol-induced ulcer model was used to evaluate the gastroprotective potential of **ALI**. This model has been widely used to study the efficacy of crude extracts and compounds with putative gastroprotective effect. Lesions produced by ethanol include solubilization of the protective mucus and exposure of the gastric mucosa to hydrochloric acid (HCl) and proteolytic enzymes, which, in turn, lead to plasma membrane damage and permeability alterations. Ethanol also stimulates HCl secretion, decreases blood flow to the epithelium, increases the production of reactive species derived from oxygen (ROS) and alters bicarbonate secretion. The advantages of using this model are that the lesions caused by the acute administration of ethanol are highly reproducible, in addition to the fact that the alterations generated closely resemble those of human peptic ulcers [38,39]. The assessment of the effect of **ALI** on acute gastric ulcers revealed important protective activity against ethanol. The maximal gastroprotective effect exerted by **ALI**, i.e., 69.8% at 300 mg/kg, was comparable with that of carbenoxolone at 50 mg/kg. These results are supported by the macroscopic observation of the stomachs, in which **ALI** exhibited an important reduction in the length, width and number of gastric ulcers compared with the vehicle (negative control). It is possible that by using a higher dose of **ALI**, an increment in the percentage of gastroprotection could be achieved. It is worth highlighting the fact that this is the first report on the antiulcer activity of **ALI**.

An inflammatory component constitutes a key factor in the pathogenesis of gastritis and gastric ulcers associated with *H. pylori* infection. The colonization of the gastric mucosa by *H. pylori* activates a set of signaling cascades that, in turn, provoke the transcription and translation of the relevant inflammatory mediators, such as IL-8, IL-6, INF-γ, TNF-α, COX-2, and ICAM-1, thus triggering further inflammatory responses. Moreover, chronic inflammation can lead to different pathologies, ranging from atrophic gastritis to adenocarcinoma [40]. As part of an integral evaluation of the aqueous extract of *A. ludoviciana* (**ALI)**, a preliminary study on its anti-inflammatory potential was conducted, using the well-known xylene-induced mouse ear edema in vivo model [41]. In this acute test, xylene instantaneously induces a neurogenic edema, partially due to a neurotransmitter known as substance P, which causes vasodilation and plasma extravasation, followed by the release of inflammatory mediators such as histamine, kinins and fibrinolysin. In the present work, the topical and oral administration of **ALI** provoked a moderate anti-inflammatory effect in mice without any difference between the two routes of administration. These results comprise the second report on the anti-inflammatory effect of *A. ludoviciana* aqueous extract. Similar findings were reported by Rivero-Cruz et al. [36], who described the antinociceptive and anti-inflammatory activities of an *A. ludoviciana* aqueous extract and two major sesquiterpene-type compounds, i.e., achillin and dehydroleucodin, using the formalin- and carrageenan-induced paw edema tests. In the first model, the subcutaneously administered extract showed a significant antinociceptive effect during the inflammatory phase (25 and 63% with 100 and 316 mg/kg, respectively); in contrast, in the carrageenan-induced edema, oral administration of 100 mg/kg aqueous extract reduced edema by 83% within the first hour, but this value was reduced to 67% at the third hour, with respect to the control.

Even though the anti-inflammatory mechanism of the *A. ludoviciana* extract is not entirely clear, in a work performed with an ethanol extract, it was suggested that it may have been due to interference in NF-κB activation and IL6 transcription [42].

In summary, from the results obtained with **ALI**, it is noteworthy that the extract seems to exhibit polypharmacological properties.

In other species belonging to the Artemisia genus, anti-inflammatory and gastric anti-ulcer effects have been evaluated in different models. For instance, gastroprotection has been reported for the aqueous extracts of *A. absinthium* (91% with 400 mg/kg) [43], *A. capillaris* (85% with 200 mg/kg) [44] and a pectic fraction of an aqueous extract from *A. campestris* (77% with 0.3 mg/kg) [45]. Their effect was attributed to their antioxidant action and anti-inflammatory capacity, and to the improvement of gastric mucosa protective mechanisms [43,44,45].

With respect to the anti-inflammatory potential, the intraperitoneal administration of *A. dracunculus* ethanolic extract produced a dose-dependent effect in xylene-treated mice, with an ED_50_ = 78.20 mg/kg [46]. Moreover, a 70% ethanolic extract of *A. capillaris*, exhibited an in vitro inhibition of 5-lypoxygenase, an enzyme that produces leukotrienes (inflammatory mediators) from arachidonic acid. Meanwhile, in the arachidonic acid-induced ear edema model in mice, an anti-inflammatory effect (60%) was observed at a dose of 100 mg/kg [47]. A more in-depth study was performed with an ethanol extract of *A. asiatica*. The authors reported 64% gastroprotection with 100 mg/kg of the extract, an anti-inflammatory response explained by a decrease of pro-inflammatory cytokines (IL-1β and IFN-γ) and an increase of the anti-inflammatory cytokine IL-10. Moreover, antioxidative actions were validated by an attenuation of lipid peroxidation and a minor depletion of GSH [48].

Taking into account the fact that **ALI** exhibited good anti-*H. pylori*, antiulcerogenic and anti-inflammatory activities, a preliminary fractionation was performed with the aim of reducing its complexity and exploring the possibility of improving its effects. The anti-*H. pylori* activity (Table 1) was mainly enhanced with **ALI-1** (MIC = 62.5 μg/mL), and to a lesser extent, with **ALI-2** (dichloromethane and ethyl acetate fractions, respectively) compared with **ALI**. Moreover, both fractions were found to be more active than the reference antibiotic, metronidazole (MIC = 300 μg/mL). These data indicate that the compounds exerting the inhibitory effects upon *H. pylori* are of low and intermediate polarity. The gastroprotective activity was significant with the ethyl acetate fraction **ALI-2** (86.3% with 100 mg/kg), improving the activity of **ALI** by almost two fold at the same dose. Additionally, the gastroprotection attained was even better than with **ALI** at the highest tested dose (300 mg/kg, Figure 1). The other tested fractions slightly improved the effect compared with **ALI,** but were not statistically significant (*p* > 0.05). The effect of **ALI-2** was as good as that of carbenoxolone (78.2 ± 3.5% at 50 mg/kg). None of the tested fractions improved the anti-inflammatory activity with respect to **ALI**; however, the activity was maintained with some of the evaluated fractions, such as **ALI-3** by topical administration, and **ALI-2**, **3** and **4** by oral administration. Indeed, the anti-inflammatory effect of the latter three fractions seemed to be better with oral administration compared with the topical route; this may have been due to higher dosing, but also to the pharmacokinetics of the compounds contained in the fractions [49]. In summary, these results indicate that **ALI-1** and **ALI-2** could be a potential source for either anti-*H. pylori* or gastroprotective novel compounds.

Considering our interest in finding anti-*H. pylori* agents, a bio-guided fractionation from **ALI-1** and **ALI-2** was performed. The results led us to isolate and identify two known compounds, namely, the sesquiterpene lactone estafiatin and the flavone eupatilin. Both compounds have already been reported for *A. ludoviciana* [22,50].

Eupatilin has been isolated from a variety of medicinal plants, especially from the Artemisia genus. Several studies have provided evidence of a broad spectrum of pharmacological properties such as anticancer, antioxidant, anti-inflammatory, neuroprotective, antiallergic and cardioprotective, as recently reviewed by Nageen et al. [51]. Regarding its antimicrobial activity, it seems that eupatilin did not have any antimicrobial effect on various bacteria or fungi [52].

Unlike eupatilin, estafiatin has been less widely studied. It has been reported that it may have antihypertensive effects via the inhibition of plasma angiotensin I-converting enzyme (ACE) activity [53], and even immunotherapeutic properties [54]. 

In this work, estafiatin showed better MIC than eupatilin; however, the two compounds turned out to be more effective than metronidazole, but not better than clarithromycin. Moreover, as well as stopping bacterial growth (good MIC values), both compounds also showed very good bactericidal activity (MBC) (Figure 3). Again, estafiatin was more potent than eupatilin, since it required an exposure of 14 h with 15.62 µg/mL to achieve the bactericidal effect; however, this time was reduced to only 2 h at 62.5 µg/mL. On the other hand, eupatilin achieved a bactericidal effect in 24 h with 31.25 µg/mL and in 8 h with 125 µg/mL. Once more, both compounds were much better than metronidazole, but not better than clarithromycin. To our knowledge, this work is the first report about the anti-*H. pylori* activities of estafiatin and eupatilin. The potential of these compounds as antibiotics for the eradication of *H. pylori* is encouraging, since they are new molecules which may have novel mechanisms of action, and for which resistance has not yet occurred.

Natural isolated sesquiterpene lactones from Artemisia species have shown strong antimicrobial activity against both standard and clinical isolated strains of *H. pylori*. Dehydroleucodine had MIC values in the range of 1–8 µg/mL [55]. On the other hand, Goswami et al. [56] found that artemisinin exhibited MIC_90_ and MBC_50_ values of 8 µg/mL, while artemisinic acid was less active, with MIC_90_ and MBC_50_ values of ≥128 µg/mL. These authors also found that some synthetic derivatives from artemisinin were even more potent than the natural product.

With the objective of exploring the possible mechanism involved in the anti-*H. pylori* activity of estafiatin and eupatilin, their effect was assessed on the enzyme urease, a very important survival and virulence factor of *H. pylori*. The results showed that the antibacterial effect does not rely on the inhibition of urease. Thus, the main antibacterial target of these compounds is not urease, and other mechanisms should be further investigated. 

Considering the knowledge that the groups of chemical molecules (i.e., flavones and sesquiterpene lactones) to which eupatilin and estafiatin belong have a wide variety of biological and pharmacological activities, it is certain that the isolated compounds from *A. ludoviciana* should be considered in further studies as potential constituents of therapies against the diseases related to *H. pylori* infection. In particular, it will be interesting to assess their toxicity, acid stability, in vivo efficacy and possible synergic effects with current anti-*H. pylori* antibiotics. Furthermore, it is clear that further work will be required to evaluate their polypharmacological potential, that is, not only their anti-*Helicobacter pylori* activity, but also their potential gastroprotective and anti-inflammatory effects.

## 4. Materials and Methods

### 4.1. Reagents and General Experimental Procedures

All reagents used in this study were purchased from Sigma-Aldrich (St Louis, MO, USA). Reagent grade Organic solvents were from J.T Baker. Analytical Thin-layer Chromatography (TLC) was performed on silica gel 60 F254 aluminum plates (Merck-Millipore KGaA, Darmstadt, Germany). Column chromatography (CC) was accomplished using silica gel 60 (0.2–0.5 mm) and mesh 3.5 to 7 ASTM (Merck-Millipore KGaA, Darmstadt, Germany). TLC visualization was accomplished with either a UV lamp or a ceric ammonium sulfate solution.

### 4.2. Plant Material

Aerial parts of *Artemisia ludoviciana* subsp. *mexicana* (Willd. Ex Spreng.) D.D. Keck were acquired fresh at a local market (Mercado de Sonora, Mexico City, Mexico). Plant material was identified by Edelmira Linares and Robert Bye from the Biology Institute of Universidad Nacional Autónoma de México (UNAM). A voucher specimen was deposited in the National Herbarium of Mexico (MEXU 1259898). The plant name was checked in the Tropicos-Missouri Botanical Garden database [57], and is an accepted name in The Plant List. Aerial parts from the plant were air-dried at room temperature and ground to a fine powder using an electric mill (Thomas 8455.6 Wiley, Thomas Scientific, Swedesboro, NJ, USA).

### 4.3. Extract Preparation and Primary Fractionation

*Artemisia ludoviciana* aqueous extract (**ALI**) was prepared by infusion. Briefly, 50 g of pulverized dried plant material was mixed with 1.25 L of recently boiled water (93 °C at 2200 m above sea level, Mexico City, Mexico). After 30 min, the infusion was filtered. The extraction procedure was carried out until 1.5 kg of dry plant material had been processed, which was enough for the characterization of **ALI** and the primary fractions, as well as for the isolation of the compounds used in the present study. **ALI** was subjected to a liquid–liquid extraction process using different solvents (i.e., dichloromethane, ethyl acetate, and *n*-butanol) in a 1:1 ratio. Organic phases and the aqueous residue were evaporated to obtain the four primary fractions. The abbreviations “**ALI-1**” to “**ALI-4**” were used to identify the dichloromethane, ethyl acetate, *n*-butanol, and residual extracts, respectively.

### 4.4. Isolation and Identification of Compounds

The ^1^H- and ^13^C-Nuclear Magnetic Resonance (NMR) spectra of estafiatin were recorded using a Varian Inova spectrometer (Varian Inc., Palo Alto, CA, USA), (500 and 125 MHz, respectively) at the Chemistry Institute of UNAM, Mexico City, Mexico. The ^1^H- and ^13^C-NMR spectra of eupatilin were recorded on a Varian/Agilent spectrometer (AR Premium COMPACT; Santa Clara, CA, USA) (600 and 151 MHz, respectively) at a Cinvestav facility in Mérida, Yucatán. For both compounds, deuterated chloroform (CDCl_3_) and tetramethylsilane (TMS) were used as the solvent and as internal reference, respectively. Chemical shifts are listed as δ values in ppm.

#### 4.4.1. Isolation and Identification of Estafiatin

The active primary fraction **ALI-1** (6.77 g) was applied to a silica gel CC (180.2 g) and eluted with different solvent mixtures (hexane/dichloromethane and dichloromethane/methanol). Two hundred and six secondary fractions were obtained. These fractions were then pooled into 17 final secondary fractions (**ALI-1**-I to XVII) according to their thin-layer chromatographic profiles. From the secondary fraction VIII, a crystalline translucent solid (110.8 mg) spontaneously precipitated, with a melting point of 104 °C. A sample of 10 mg was used to determine the ^1^H and ^13^C spectra; an analysis and comparison of the spectra with those previously reported data in the literature [53] allowed us to identify this compound as estafiatin.

Estafiatin (**a**): ^1^H-NMR (500 MHz, CDCl_3_). δ (ppm) 6.20 (d, 1H, *J* = 3.5 Hz, H-13a), 5.48 (d, 1H, *J* = 3.5 Hz, H-13b), 4.94 (s, 1H, H-14a), 4.85 (s, 1H, *J* = 1.5 Hz, H-14b), 4.06 (dd, 1H, *J* = 10.5 Hz, *J* = 9.15 Hz, H-6), 3.36 (s, 1H, H-3), 2.96 (td, 1H, *J* = 11 Hz, *J* = 8 Hz, H-1), 2.86 (m, 1H, H-7), 2.30 (dd, 1H, *J* = 11, *J* = 8 Hz, H-5), 2.20 (m, 1H, H-9a), 2.06 (dd, 1H, *J* = 14 Hz, *J* = 7.5 Hz, H-2a), 1.80 (dd, 1H, *J* = 14 Hz, *J* =11.5 Hz, H-2b), 1.60 (s, 3H, H-15), 1.52 (m, 1H, H-9b). ^13^C-NMR (125 MHz, CDCl_3_). δ (ppm) 169.8 (C-12), 146.3 (C-10), 139.7 (C-11), 120.3 (C-13), 115.4 (C-14), 80.7 (C-6), 66.0 (C-4), 63.3 (C-3), 51.0 (C-5), 45.0 (C-1), 44.3 (C-7), 33.1 (C-2), 29.4 (C-9), 28.8 (C-8), 18.7 (C-15).

#### 4.4.2. Isolation and Identification of Eupatilin

Active primary fraction **ALI-2** (1.40 g) was subjected to a secondary fractionation on a silica gel CC (150 g) and eluted using different mixtures of hexane/ethyl acetate and ethyl acetate/methanol. As a result, 208 secondary fractions were obtained; after a TLC analysis, these fractions were pooled into six secondary fractions (**ALI-2**-A to F). From the active secondary fraction C, 181.4 mg was separated by molecular exclusion chromatography using Sephadex^®^ LH-20 (spheres 25–100 μm) and methanol as the mobile phase. Through this process, five fractions (C.1 to C.5) were obtained. A yellow solid was obtained from C.4 fraction (102.1 mg) which, after comparison of the NMR spectroscopy data with those reported for this compound [58], was identified as the flavonoid eupatilin.

Eupatilin (**b**): ^1^H-NMR (600 MHz, CDCl_3_). δ (ppm) 13.06 (s, 1H), 7.52 (dd, 1H, *J* = 8.5, 2.0 Hz), 7.33 (d, 1H, *J* = 1.9 Hz), 6.98 (d, 1H, *J* = 8.5 Hz), 6.59 (d, 2H, *J* = 16.2 Hz), 4.04 (s, 3H), 3.98 (s, 3H), 3.96 (s, 3H). ^13^C-NMR (151 MHz, CDCl_3_). δ (ppm) 183.03 (C-4), 164.22 (C-2), 155.14 (C-9), 153.26 (C-5), 152.45 (C-7), 152.26 (C-4′), 149.47 (C-3′), 130.48 (C-6), 123.90 (C-1′), 120.25 (C-6′), 111.32 (C-5′), 108.93 (C-2′), 105.88 (C-3), 104.21 (C-10), 93.50 (C-8), 61.03 (6-OCH_3_), 56.26 (3′, 4′-OCH_3_).

### 4.5. Bacterial Strain and Culture Conditions

*H. pylori* (ATCC 43504) was cultured on Casman agar base, supplemented with 5% defibrinated sheep blood, 10 mg/L vancomycin, 5 mg/L trimethoprim, 2 mg/L amphotericin B and 2.5 mg/L polymyxin B at 37 °C for 24 h under microaerophilic conditions (10% CO_2_, 5% O_2_, 85% N_2_). Stock cultures were stored at −70 °C in Brucella broth, supplemented with 10% fetal bovine serum (GIBCO BRL) and 10% glycerol. The strains were routinely identified by Gram staining, morphology and biochemical testing.

### 4.6. Anti-Helicobacter pylori Activity

The antibacterial activity was evaluated according to the Clinical and Laboratory Standards Institute (CLSI) guidelines [59], by the broth dilution method. Bacteria were grown on Mueller–Hinton broth (DIFCO, Becton Dickinson and Company, Sparks, MD, USA), with 0.2% β-cyclodextrin, vancomycin (10 mg/L), trimethoprim (5 mg/L), amphotericin B (2 mg/L) and polymyxin B (2.5 mg/L), and incubated under gentle shaking (150 rpm) at 37 °C under microaerophilic conditions (10% CO_2_, 5% O_2_, 85% N_2_). Different concentrations of either **ALI** fractions **ALI-1**, **ALI-2**, **ALI-3** or **ALI-4** (7.8 to 250 μg/mL), or compounds (7.8 to 125 μg/mL), dissolved in DMSO, were added in a volume of 10 μL to 1.5 mL *H. pylori* broth culture at the beginning of the exponential phase (~10^8^ CFU/mL). The difference of absorbance at 600 nm was determined after 24 h of incubation (10% CO_2_, 37 °C and 150 rpm); this was then used to calculate the percentage of growth inhibition vs. the control (10 μL DMSO, which did not have any effect on bacterial growth). The minimum inhibitory concentration (MIC) was defined as the lowest concentration of the extract or compound that completely inhibited bacterial growth.

In order to determine the bactericidal effect, samples from the broth cultures exposed to different concentrations of the compounds were taken at various growing times, and the bacterial viability was assessed by the plate colony count technique. Serial 10-fold dilutions were made with Brucella broth and plated onto Casman agar plates. Colony counting was performed after 7 days of incubation at 37 °C under microaerophilic conditions. Minimum bactericidal concentration (MBC) was defined as the lowest compound concentration that killed all bacteria within a defined period of time. All experiments were carried out in triplicate and repeated at least three times. Clarithromycin (0.05 μg/mL) and metronidazole (300 μg/mL) were used as positive controls.

### 4.7. Urease Inhibition Assay

Urease was obtained from a liquid culture of *H. pylori* at the logarithmic phase of growth. Bacteria were washed twice with PBS pH 7.2 and then sonicated in the presence of a protease inhibitor cocktail (complete Roche™, Roche Applied Science, Mannheim, Germany). The lysate was then centrifuged at 17,000× *g* for 10 min at 4 °C. The supernatant was collected and ultracentrifuged at 130,000× *g* for 50 min at 4 °C. The resulting supernatant from this last centrifugation was used as the urease sample. Aliquots of the enzyme were stored at 4 °C prior to use. Protein concentration was quantified by Bradford’s method using BSA as standard [60].

Urease activity was determined by measuring the NH_4_^+^ released by the hydrolysis of urea using Berthelot’s method with some modifications [61]. The reaction medium contained either 5 µL of DMSO, eupatilin or estafiatin (3.9, 7.81, 15.62, 31.25, 62.5 y 125 μg/mL) freshly dissolved in this solvent, 3 μg protein of *H. pylori* urease and PBS in a final volume of 150 µL. This mixture was pre-incubated for 5 min. The reaction was started by adding urea (5 mM final concentration) and incubated for 10 min at 37 °C. For the colorimetric reaction, 50 µL of 354 mM phenol and 100 µL of 345 mM NaOH/176 mM NaClO were added and the absorbance at 600 nm was recorded after 5 min incubation at room temperature using a Bio-Rad 2550 EIA Reader (Bio-Rad Laboratories, Inc., Pleasanton, CA, USA). Ammonium sulfate was used as standard for the calibration curve. The percentage of enzymatic inhibition was calculated with respect to the activity without an inhibitor. Acetohydroxamic acid (AHA) was used as a positive control. At least three independent experiments were performed in triplicate.

### 4.8. Animals

Experiments were conducted on adult male CD-1 mice provided by UNAM’s Facultad de Medicina vivarium, Mexico City, Mexico. Mice weighing 40–45 g were used for the evaluation of gastric antiulcerogenic activity, whereas mice weighing 20–25 g were used for anti-inflammatory and acute toxicity assays. Animals were housed in a temperature-controlled facility (21 ± 2 °C) with a 12/12 h light-dark cycle and with free access to water and standard rodent chow. The experimental protocols were approved by the Ethics Committee for the Care and Use of Laboratory Animals, Faculty of Medicine, UNAM, Mexico City, Mexico (CICUAL 075/2018), and were conducted in accordance with the Mexican Official Norm of Animal Care and Handling and in compliance with the international guidelines on ethical standards for investigations on animals.

### 4.9. Acute Oral Toxicity Assay

Lorke’s method [62] was used to determine the acute toxicity effect of **ALI**, the *A. ludoviciana* aqueous extract. ALI was suspended in PBS and the concentrations were adjusted for oral administration at 0.2 mL/10 g body weight. Mice were weighed, randomly divided into control and experimental groups (*n* = 3) and treated in two independent phases. In the first phase, the control (vehicle) and experimental groups received 10, 100 and 1000 mg/kg of the corresponding treatment, and were observed for 24 h to monitor their behavior or mortality. Based on the results of the first phase, we proceeded to the second phase in which new animal groups (*n* = 3) were administered higher doses (1600, 2900 and 5000 mg/kg) of the tested substance and then observed for 24 h for behavioral changes or mortality. Mice were monitored for a 14-day period to determine any abnormal behavior, toxicity signs or mortality. At the end of the testing period, surviving animals were euthanized in a CO_2_ chamber. LD_50_ was calculated as described by Lorke.

### 4.10. Gastric Antiulcerogenic Activity

Ethanol-induced gastric ulceration was performed according to the method described by Bucciarelli [38]. Mice were divided into different groups (*n* = 7). Each group received either vehicle (7 mL/kg), **ALI** (10–300 mg/kg), **ALI-1**, **ALI-2**, **ALI-3** or **ALI-4** (100 mg/kg), or carbenoxolone (CAR) 50 mg/kg as a positive control, by gastric gavage. CAR and samples were freshly prepared and dissolved in saline solution (0.9% NaCl). After 1 h, all groups were orally administered a dose of 7 mL/kg body weight (BW) with absolute ethanol to induce gastric ulceration. One hour and a half after ethanol administration, mice were sacrificed in a CO_2_ chamber. Each stomach was dissected out, insufflated with 2 mL of 2% formalin and fixed in the same solution for 15 min. The stomachs were opened along the greater curvature, pressed between two glass plates, and scanned. Gastric lesions were assessed with the aid of a public domain Java image processing program (ImageJ) developed at the US National Institute of Health. The sum of the areas of all lesions in the corpus of each stomach was calculated and considered as the ulcer area.

### 4.11. Anti-Inflammatory Activity

Acute inflammation was evaluated by the xylene-induced ear edema model, as described by Okoli et al. [63] and Eddouks et al. [41], with some modifications. The anti-inflammatory effect of **ALI** and the four primary fractions (**ALI-1** to **ALI-4**) was tested by topical and oral administration. Briefly, under general anesthesia with sodium pentobarbital (60 mg/kg intraperitoneally), 0.5 mg/ear of the samples (dissolved in 20 μL of 70% ethanol) or the positive control, indomethacin (dissolved in 20 μL of acetone), were topically applied in the right ear. Next, local inflammation was induced by applying the irritant agent (30 μL of xylene) in the same ear. The left ear, considered as the control, received the same volume of the corresponding vehicle. For the oral treatment, 30 min before receiving the irritant agent, saline solution, indomethacin (30 mg/kg) or the samples (100 mg/kg) were administered via the intragastric route at a volume of 7 mL/kg BW. One hour after xylene-induced inflammation, animals were sacrificed in a CO_2_ chamber; circular sections of 7 mm diameter were removed from both treated and untreated ears and weighed. The percentage of edema was calculated based on the weight of the left ear (without xylene) using the following formula:

$$\%\;edema\;inhibition=\left[\frac{\Delta \;ear\;edema\;control-\Delta \;ear\;edema\;treated}{\Delta \;ear\;edema\;control}\right]\times 100$$

### 4.12. Statistical Analysis

Comparisons regarding dose- or concentration-responses were analyzed by one-way analysis of variance (ANOVA) followed by Dunnett’s post hoc test. All data are expressed as the mean ± SEM. *P* values < 0.05 were considered statistically significant. All analyses were performed using the Graph Pad Prism software version 8.0 (San Francisco, CA, USA).

## 5. Conclusions

With the objective of establishing the efficacy of *Artemisia ludoviciana* subsp. *mexicana* as a treatment for *H. pylori*-related diseases, in this study, an infusion (**ALI**) and four primary fractions (**ALI-1** to **4**) from aerial parts of this species were tested for potential polypharmacological effects.

The findings showed that **ALI** has significant anti-*H. pylori*, gastroprotective and anti-inflammatory activities. This information comprises a remarkable contribution to the ethnopharmacological knowledge of this species, since aqueous preparations are the most common form in which the plant is used to treat digestive disorders. Our study therefore validates the traditional consumption methods of *Artemisia ludoviciana* subsp. *mexicana*. 

Many studies have shown that different plant extracts or compounds could aid in *H. pylori* eradication, or have promising therapeutic effects on gastric ulcers. **ALI** was shown to possess these properties. Considering the problems associated with current therapies for *H. pylori*-associated diseases, the development of treatments based on natural extracts with polypharmacological properties is encouraging due to the synergistic interactions between its components, thereby reducing the risk of resistance to a specific drug. Therefore, *A. ludoviciana* is emerging as a candidate for the development of an integral therapy.

Finally, the roles of estafiatin and eupatilin (isolated from *A. ludoviciana* infusion) as antibiotics against *H. pylori* were demonstrated for the first time. This opens a very important research path for their possible future therapeutic use.

## Figures and Tables

**Table 1:** Anti-*H. pylori*, gastroprotective and anti-inflammatory effects of the aqueous extract and organic fractions from *A. ludoviciana*.

		Activities			
Extract and Fractions	Yield (%)	Anti-*H. pylori*	Gastroprotective %	Anti-Inflammatory %	
		MIC ^d^ (μg/mL)	Oral	Topical	Oral
			100 mg/kg	0.5 mg/ear	100 mg/kg
Aqueous (**ALI**)		250	49.6 ± 7.5	38.8 ± 10.2	47.6 ± 12.4
Dichloromethane (**ALI-1**)	7.9	62.5	61.0 ± 5.2	23.4 ± 6.6	20.7 ± 4.1
Ethyl acetate (**ALI-2**)	8.5	125	86.3 ± 3.9	29.4 ± 10.8	47.2 ± 5.3
*n*-butanol (**ALI-3**)	21.4	>250	61.8 ± 11.4	31.8 ± 12.8	51.3 ± 6.7
Residual (**ALI-4**)	62.1	>250	66.9 ± 10.6	0 ± 6.6	36.5 ± 6.6
Metronidazole ^a^		300			
Clarithromycin ^a^		0.01			
Carbenoxolone ^b^ (50 mg/kg)			78.2 ± 3.5		
Indomethacin ^c^ (topical 0.5 mg/ear, oral 30 mg/kg)				61.3 ± 8.1	54.8 ± 4.7

^a^ Reference antibiotics for anti-*H. pylori* test. ^b^ Positive control for gastroprotective activity. ^c^ Positive control for anti-inflammatory activity. ^d^ MIC: Minimum inhibitory concentration.

## Data Availability

The data presented in this study are available on request from the corresponding author.

## Supplementary Materials

The following are available online, Figures S1–S4 ^1^H-NMR and ^13^C-NMR for estafiatin (**a**) and eupatilin (**b**).

## Sample Availability

Samples of both compounds (eupatilin and estafiatin) are not available from the authors due to the low yield obtained during the compounds’ isolation.
