# Supplementary material for: Anti-Helicobacter pylori Activity of Artemisia ludoviciana subsp. mexicana and Two of Its Bioactive Components, Estafiatin and Eupatilin
Source: Molecules. 2021 Jun 15;26(12):3654. doi: 10.3390/molecules26123654 (PMC8232798; doi:10.3390/molecules26123654)
Supplement: Supplementary file 1 [file molecules-26-03654-s001.zip › molecules-1209726-supplementary.pdf]

SUPPLEMENTARY MATERIAL

Anti-*Helicobacter pylori* activity of *Artemisia ludoviciana* subsp. *mexicana* and two of its bioactive components, Estafiatin and Eupatilin

Juan Francisco Palacios-Espinosa[1], Pablo Noé Núñez-Aragón[2], Erika Gomez-Chang[2], Edelmira Linares[3], Robert Bye[3], and Irma Romero[2*]

Affiliations

[1] Departamento de Sistemas Biológicos, División de Ciencias Biológicas y de la Salud, Universidad Autónoma Metropolitana-Xochimilco (UAM-X), Ciudad de México 04960, México. jpalacios@correo.xoc.uam.mx

[2] Departamento de Bioquímica, Facultad de Medicina, Universidad Nacional Autónoma de México (UNAM), Ciudad Universitaria, Ciudad de México 04510, México. pablo.nunez@uaem.mx, ergoc@bq.unam.mx, irma@bq.unam.mx

[3] Jardín Botánico, Instituto de Biología, Universidad Nacional Autónoma de México (UNAM), Ciudad Universitaria, Ciudad de México 04510, México. mazari@ib.unam.mx, rbye@ibiologia.unam.mx

* Correspondence: irma@bq.unam.mx; Tel.: +52 55 56-23-25-11.

Supplementary material

Table of Contents			
			Page
**Figure 1S**	^{1}H NMR spectrum of Estafiatin in $CDCl_3$ (500 MHz)		3
**Figure 2S**	^{13}C NMR spectrum of Estafiatin in $CDCl_3$ (125 MHz)		4
**Figure 3S**	^{1}H NMR spectrum of Eupatilin in $CDCl_3$ (600 MHz)		5
**Figure 4S**	^{13}C NMR spectrum of Eupatilin in $CDCl_3$ (151 MHz)		6

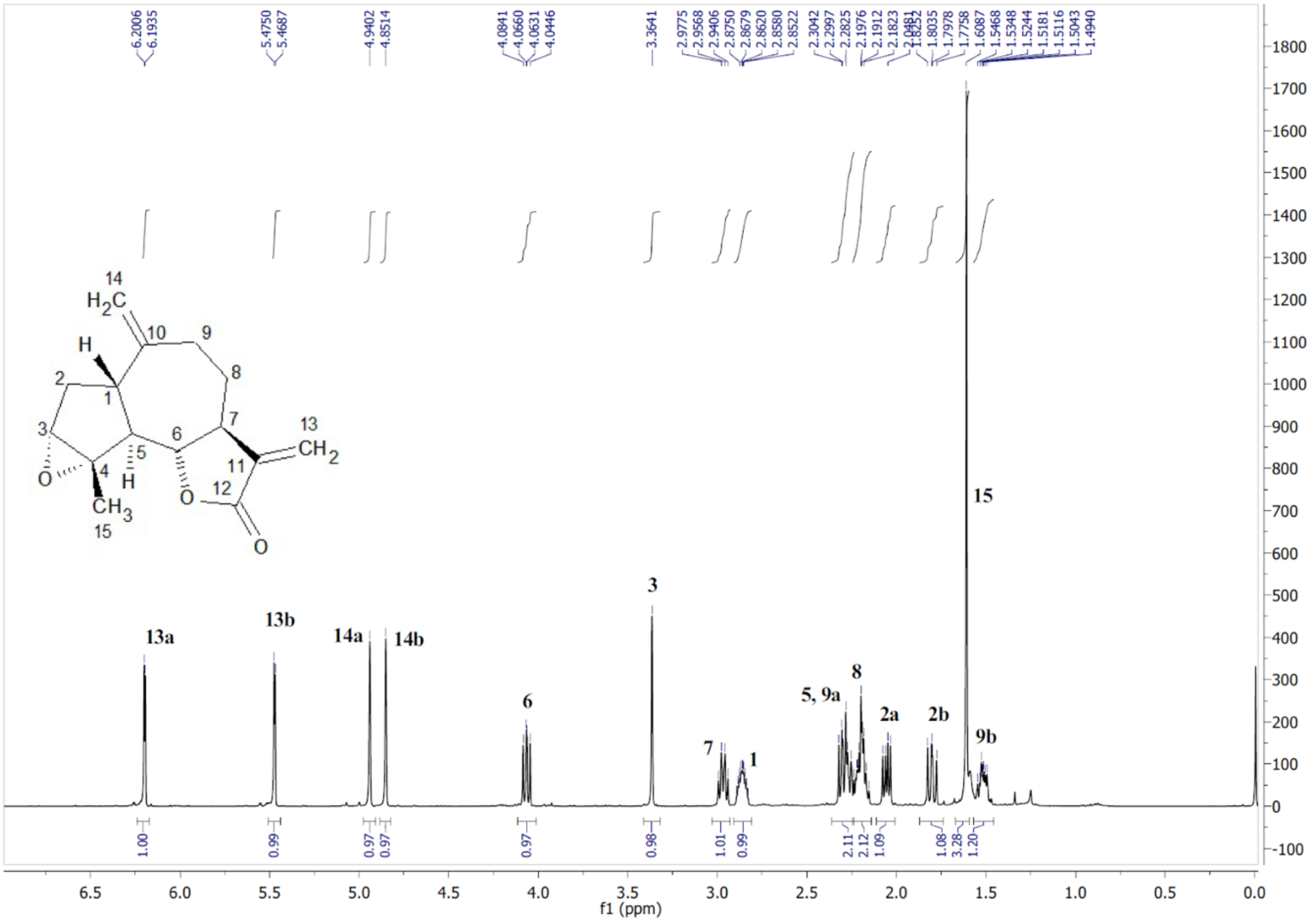

Figure 1S. ^{1}H NMR spectrum of Estafiatin in $CDCl_3$ (500 MHz).

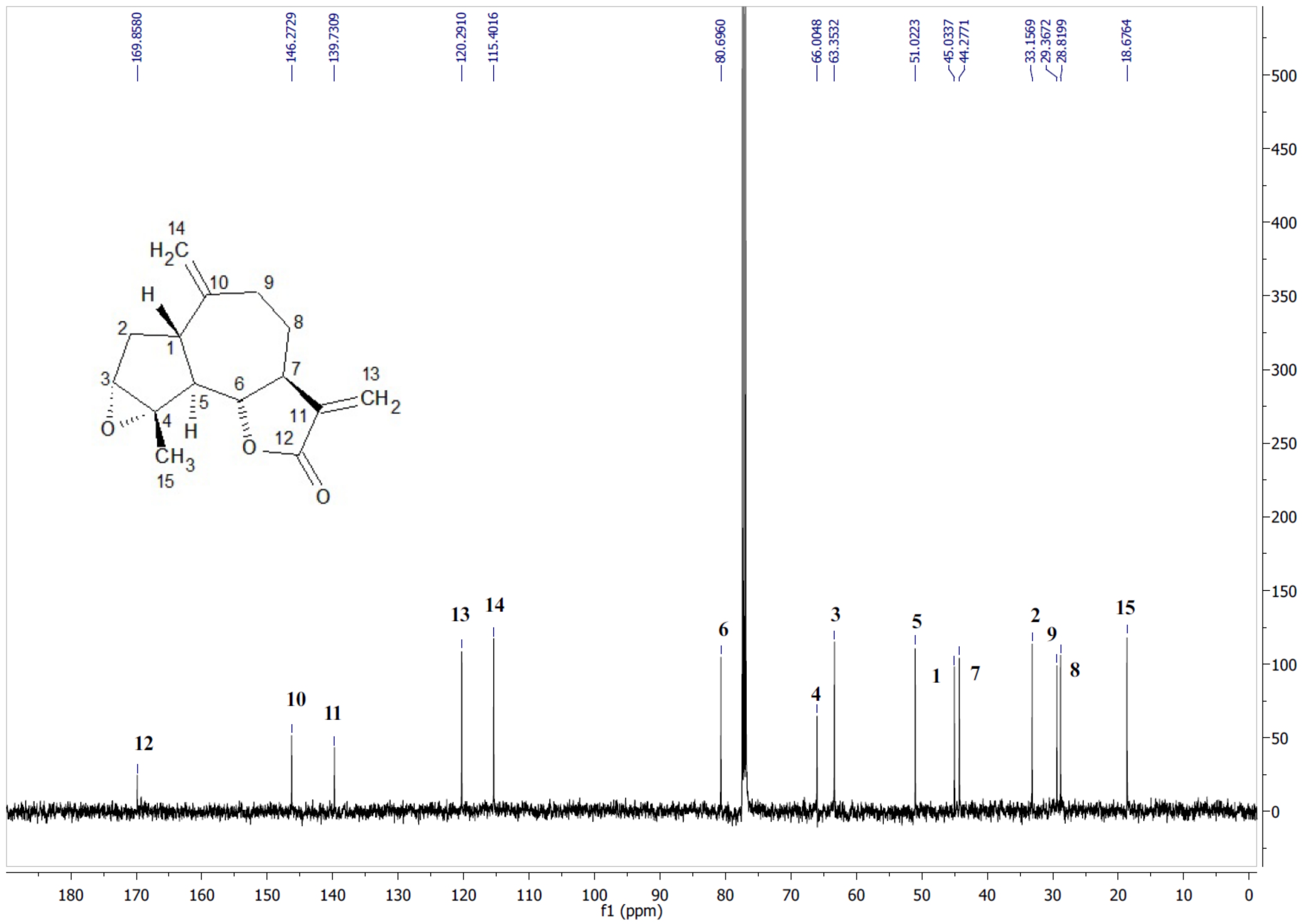

Figure 2S. ^{13}C NMR spectrum of Estafiatin in $CDCl_3$ (125 MHz).

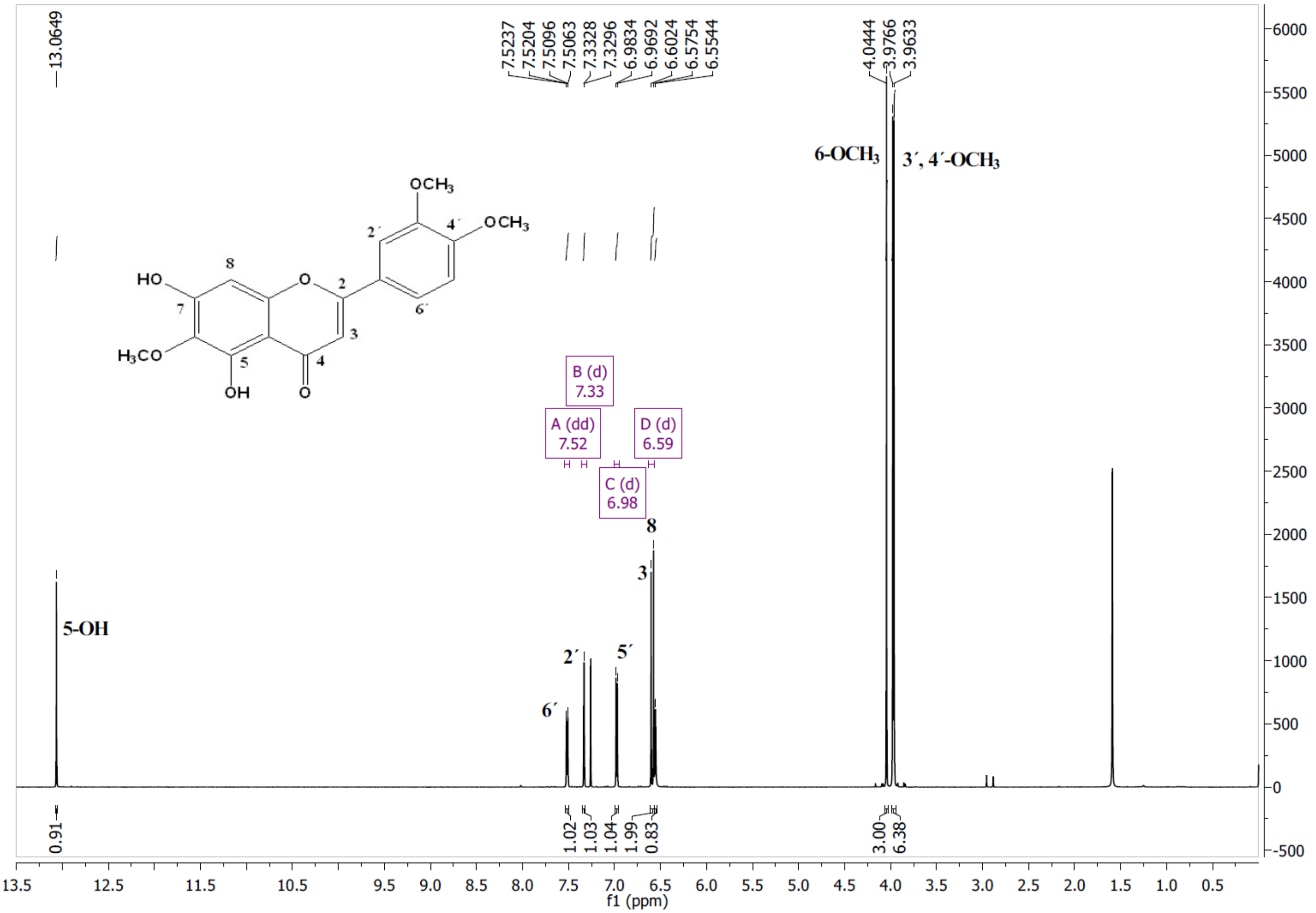

Figure 3S. ^{1}H NMR spectrum of Eupatilin in $CDCl_3$ (600 MHz).

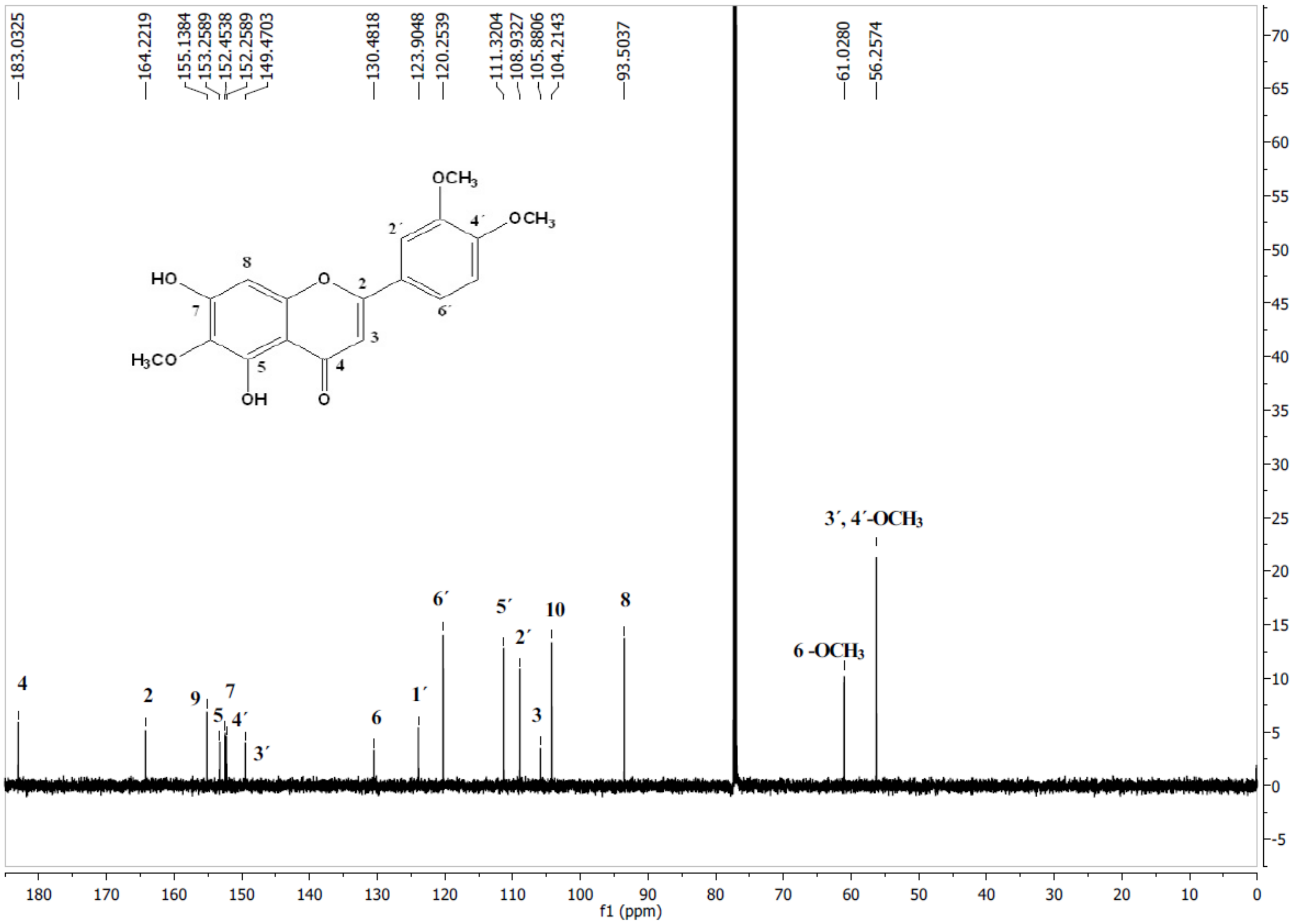

Figure 4S. ^{13}C NMR spectrum of Eupatilin in $CDCl_3$ (151 MHz).
